# THAT’S SO CRINGE: Exploring the Concept of Cringe or Vicarious Embarrassment and Social Pain

**DOI:** 10.1192/j.eurpsy.2022.1722

**Published:** 2022-09-01

**Authors:** S. Jesus, A. Costa, G. Simões, G. Dias Dos Santos, J. Alcafache, P. Garrido

**Affiliations:** Baixo Vouga Hospital Centre - EPE, Psychiatry And Mental Health Department, Aveiro, Portugal

**Keywords:** Empathy, personality trait, cringe, Theory of Mind

## Abstract

**Introduction:**

The word cringe has suffered alterations in its colloquial application, with its most recent version, adopted by generation Z and millennials, as a response to embarrassment or social awkwardness by proxy. This odd emotion is interesting in that it translates a vicarious embarrassment which is elicited whenever one is in the presence of a social blunder, public failures and threats to another’s social integrity.

**Objectives:**

The authors aim to explore the novel concept of cringe, briefly discussing what is currently known about the emotional response. A potential correlation between empathy and cringe is discussed as well as the hypothesis that certain psychiatric disorders such as personality disorder may demonstrate altered cringe responses.

**Methods:**

The authors propose a non-systematized brief literature review on works most pertinent to the topic.

**Results:**

Formal and structured studies into the concept of cringe are far and few between, however, the literature does demonstrate that, the neural pathways of how social closeness affects our experience of cringe are starting to be explored. The concept of cringe, has also been described as a vicarious social pain. Exploration into the empathy pathways and their abnormalities, may demonstrate the underlying construct of cringe. Lack of this feeling may be present in those with empathy alterations, such as is seen in antisocial personality disorder.

**Conclusions:**

Cringe is an uncomfortable feeling that surges when in the presence of someone suffering socially. Understanding this oddity may permit further understanding of empathy pathways as well as exploring the neural abnormalities of those who do not feel cringe.

**Disclosure:**

No significant relationships.

